# Intra-Operative Diagnosis of Benign Multicystic Peritoneal Mesothelioma: A Case Report of Rare Entity and Lessons Learned

**DOI:** 10.7759/cureus.60664

**Published:** 2024-05-20

**Authors:** Chih Ching Wu, Aman Bassi, Adedayo Onitilo, Rohit Sharma

**Affiliations:** 1 Surgery, Marshfield Medical Center, Marshfield, USA; 2 General Surgery, Saint Louis University School of Medicine, St. Louis, USA; 3 Hematology and Medical Oncology, Marshfield Clinic Health System, Marshfield, USA; 4 Surgical Oncology, Marshfield Medical Center, Marshfield, USA

**Keywords:** hyperthermic intraperitoneal chemotherapy (hipec), intra-abdominal tumors, cytoreductive surgery and hipec, abdominal cyst, intraabdominal tumors, multicystic peritoneal mesothelioma, benign multicystic peritoneal mesothelioma

## Abstract

Benign multicystic peritoneal mesothelioma (BMPM), also known as multicystic peritoneal mesothelioma (MCPM), is a rare cystic neoplasm arising from the mesothelium lining of the abdominal and pelvic peritoneum. This entity has been disproportionately described in women of reproductive age. Both the etiology and pathogenesis of the condition are not well understood. Preoperative diagnosis is challenging as differentials are varied and include endometriosis, lymphangioma, pseudomyxoma peritonei, cystic adenomatoid tumor, and malignant peritoneal mesothelioma. Management options include cytoreductive surgery (CRS) with or without heated intraperitoneal chemotherapy (HIPEC). In this case report, we highlight the complexity of preoperative diagnosis, presentation, workup, treatment, and management of BMPM. We report the case of a female patient presenting with abdominal pain and imagining consistent with cystic intra-abdominal lesions. After an inconclusive percutaneous biopsy and a multi-disciplinary tumor board discussion, the patient was offered CRS with HIPEC. Intra-operative frozen section indicated benign epithelial lined cysts. CRS and HIPEC were performed. After a second opinion, the lesions were confirmed by pathology and immunohistochemistry to be BMPM. In this report, we discuss the gold standard of care for patients with BMPM to improve the disease control rate. This pathway is proposed in our study, and, thus, we conclude that BMPM should be considered in the differential diagnosis of patients presenting with symptomatic multiple intraperitoneal cystic lesions.

## Introduction

Benign multicystic peritoneal mesothelioma (BMPM) is a rare tumor that disproportionately impacts females of childbearing age [1,2] and is alternatively known as “multicystic peritoneal mesothelioma,” “multiloculated peritoneal inclusion cyst,” “peritoneal mesothelial cyst,” “cystic mesothelioma,” “inflammatory cysts of the peritoneum,” or “ postoperative peritoneal cyst.” This non-metastasizing lesion originates from the mesothelium lining of the peritoneal cavity [3]. The etiology and pathogenesis of BMPM remain largely unknown, complicating its diagnosis [4]. Its differential diagnosis includes diseases with similar clinical presentation and imaging characteristics: cystic lymphangioma, endometriosis, cystadenoma/cystadenocarcinoma of the ovary, pseudomyxoma peritonei, and malignant mesothelioma [5]. Although BMPM is classified as an indolent tumor, it exhibits a high recurrence rate, with 50% of cases reoccurring 10 years after surgical resection. This necessitates long-term follow-up and a need for additional therapy [6].

We report a case highlighting the complexity of making a preoperative diagnosis, the utility of frozen sections in intra-operative decision, and the use of hyperthermic intraperitoneal chemotherapy (HIPEC) to manage the disease. We also discuss the long-term outcomes associated with the disease.

## Case presentation

**Figure 1:**
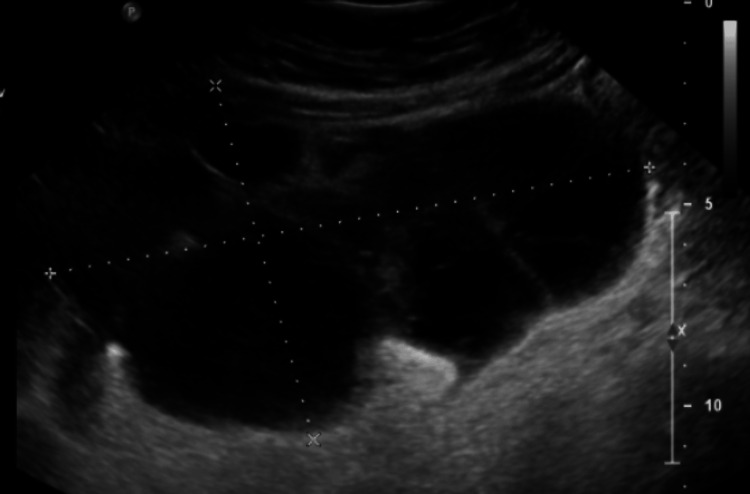
Longitudinal ultrasound of the right lower quadrant showing the septations and solid components of the adnexal mass measuring up to 15.4 cm in length. The cystic lesion appears to be associated with the ascending colon displacing loops of the small bowel.

A 43-year-old female with a past medical history of possible polycystic ovarian syndrome presented with a rapid onset of right lower quadrant abdominal pain within the last two months. She reported a progressive constant dull ache throughout her lower abdomen. She denied changes in her dietary, bowel, and urinary patterns. On physical examination, she had diffuse abdominal tenderness without evidence of peritonitis and had a palpable mass in the right lower quadrant, extending to the inferior ribs. An ultrasound showed a large right adnexal mass with septations and solid components, measuring 15.4 cm at its greatest dimension. It also showed smaller cystic areas along the medial aspect of the ovaries measuring approximately 1.5 cm to 3.2 cm (Figure 1).

**Figure 2:**
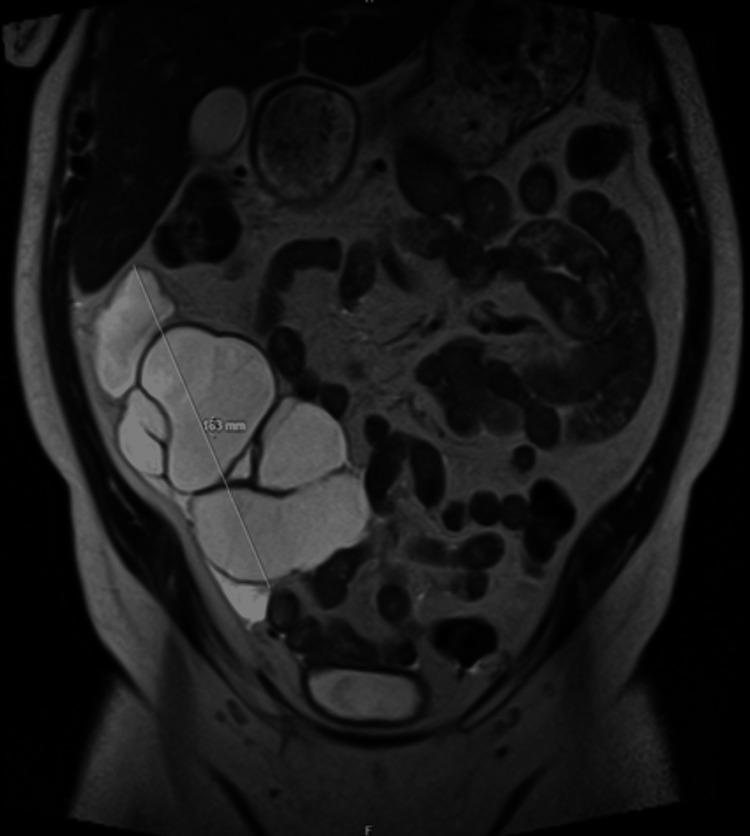
MRI showing large septated cystic lesion closely associated with the ascending colon and small bowel.

A subsequent pelvis MRI confirmed the large septated cystic lesion intimately associated with the ascending colon and that the adjacent small bowels exhibited characteristics associated serious/mucinous cystic neoplasm (Figure 2).

We anticipated the differential diagnosis of pseudomyxoma peritonei from originating from the appendix at this point. Next, we used percutaneous ultrasound-guided core needle biopsy, which showed fibroadipose tissue with lymphoplasmacytic infiltrate, histiocytes, and giant cells without evidence of mucin or malignancy. We identified three benign polyps during diagnostic colonoscopy. Her CA-125 (carbohydrate antigen) protein level was elevated at 107.7 U/mL (reference range: 0-30.2 U/mL); however, her CA 19-9 and CEA (carcinoembryonic antigen) were within normal limits (11 and <0.7, respectively) (Table 1).

**Table 1:** Patient's tumor markers prior to surgical resection. The patient had elevated CA-125 but normal CA 19-9 and CEA. CA, carbohydrate antigen; CEA, carcinoembryonic antigen

Tumor Markers	Observed Value	Reference Range
CA-125	84.4 U/mL	0-30.2 U/mL
CA 19-9	11 U/mL	<37 U/mL
CEA	<0.7 ng/mL	0-2.5 ng/mL

After surgical resection and HIPEC, her CA-125 decreased to 7.0. 

**Figure 3:**
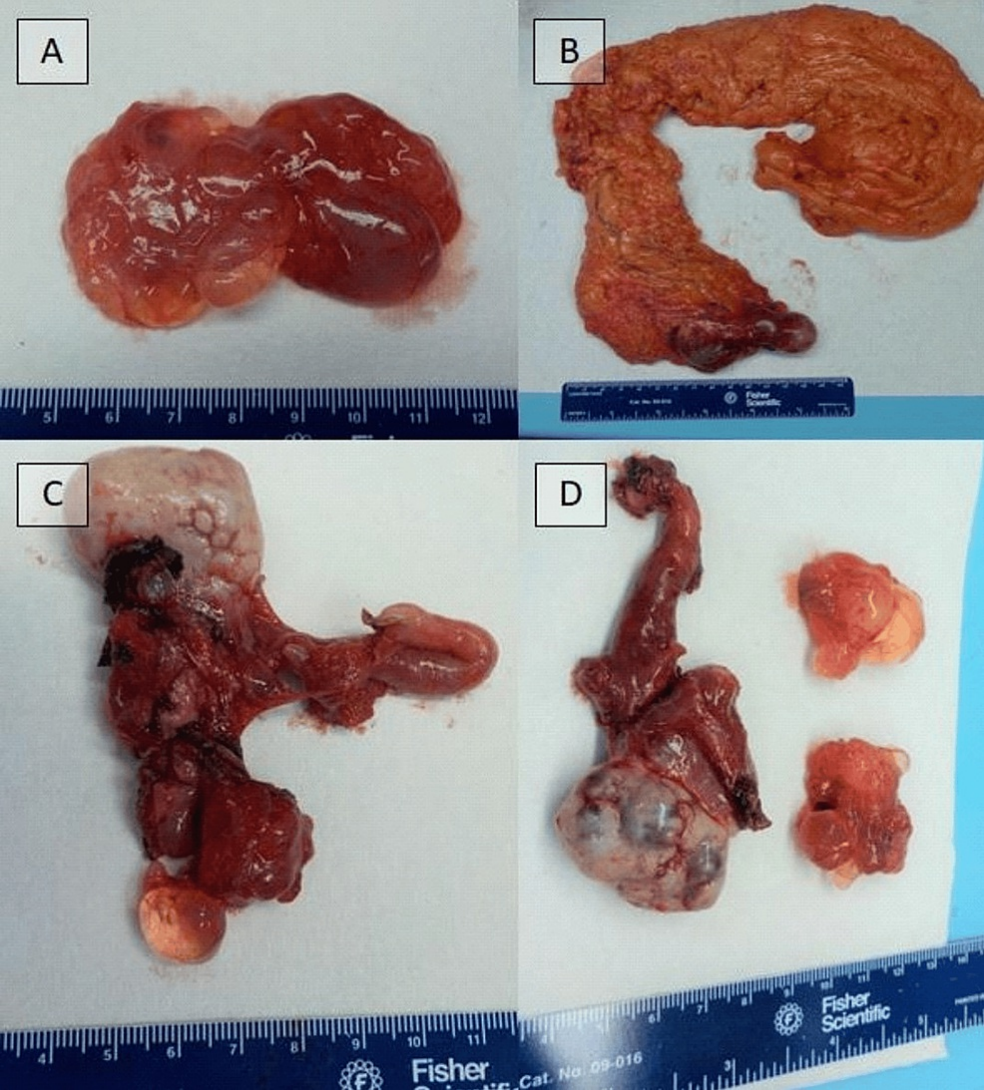
Gross specimen of cystic lesions. (A) Portions of the primary tumor showing cystic nodules. (B) Omentum. (C) Left ovary and left fallopian tube. (D) Right ovary and right fallopian tube with associated cystic lesion with similar gross characteristics to the primary tumor.

After a multi-disciplinary tumor conference discussion, the patient consented and was taken for an exploratory laparotomy with the possibility of HIPEC. Intraoperatively, multiple thin-walled, grape-like, clear fluid-containing cystic nodules were identified within the peritoneum intimately associated with various intraperitoneal organs. No ascites were noted. Next, she underwent a bilateral salpingo-oophorectomy, an omentectomy, an appendectomy, a cholecystectomy, and the resection of intra-abdominal tumors from the small bowel, pelvic side wall, and sigmoid colon. The cystic nodules were evaluated by intra-operative frozen section (Figures 3A-3D), which showed simple peritoneal cysts.

**Figure 4:**
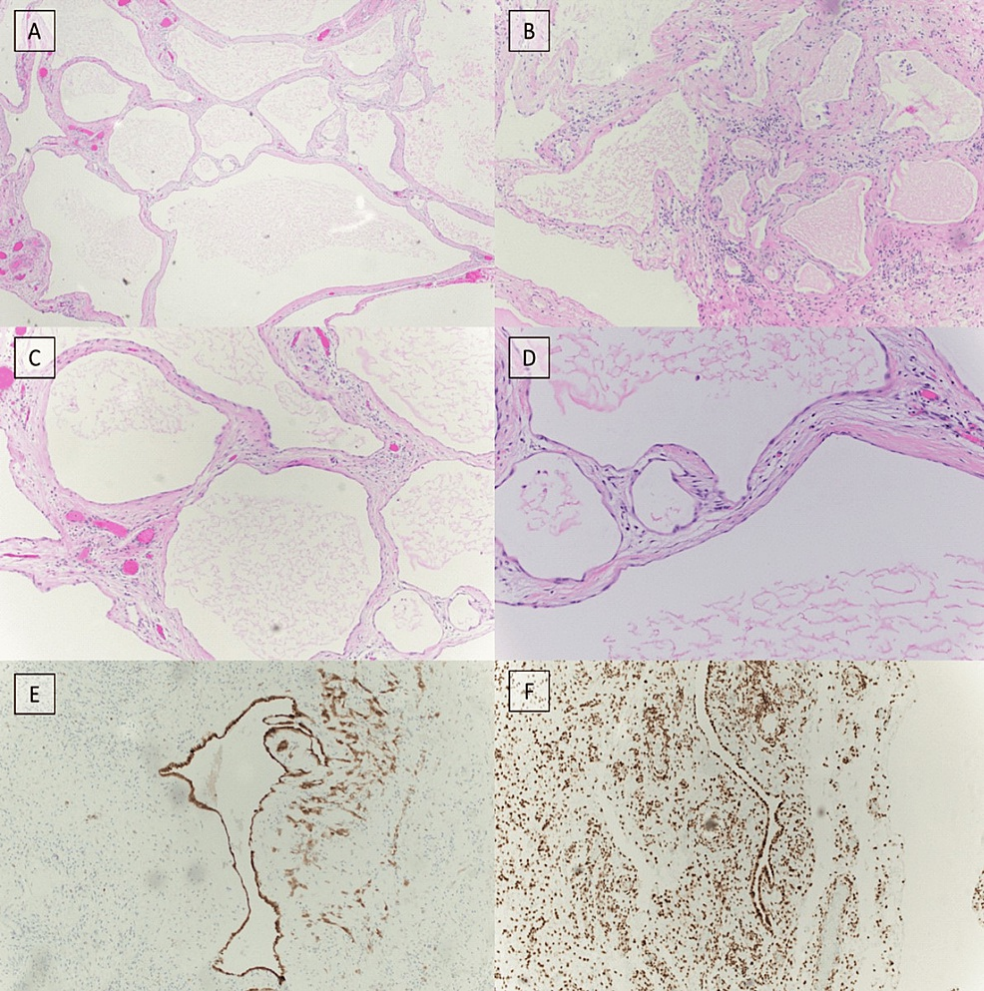
Histopathology of cystic lesions. (A) H&E stain of cystic lesion at 4x. Cysts are lined by mesothelial cells without evidence of nuclear atypia and or mitosis. Fibrous septae separate individual cysts. (B and C) H&E stain of cystic lesion at 10x. (D) H&E stain of cystic lesion at 20x. (E) Immunohistochemistry of cystic lesion with calretinin at 10x. (F) Immunohistochemistry of cystic lesion with retained BAP1 expression at 10x confirming the diagnosis of BMPM. BMPM, benign multicystic peritoneal mesothelioma; H&E, hematoxylin and eosin

This observation supported the diagnosis of BMPM. Closed circuit HIPEC was performed with 150-mg cycles of cisplatin in three equally divided doses at 0, 30, and 60 minutes for a total dwell time of 90 minutes. The target outflow temperature was above 41°C, and a flow rate of 1.5L/min was achieved. Final permanent pathology confirmed BMPM at second opinion outside the institution, with positive immunohistochemistry for calretinin, CK5/6 (cytokeratin 5/6), WT1 (Wilm's tumor 1), and BAP1 (BRCA1-associated protein-1), and negative immunohistochemistry for MOC31 (anti-epithelial related antigen) from the primary peritoneal lesion (Figure 4).

**Figure 5:**
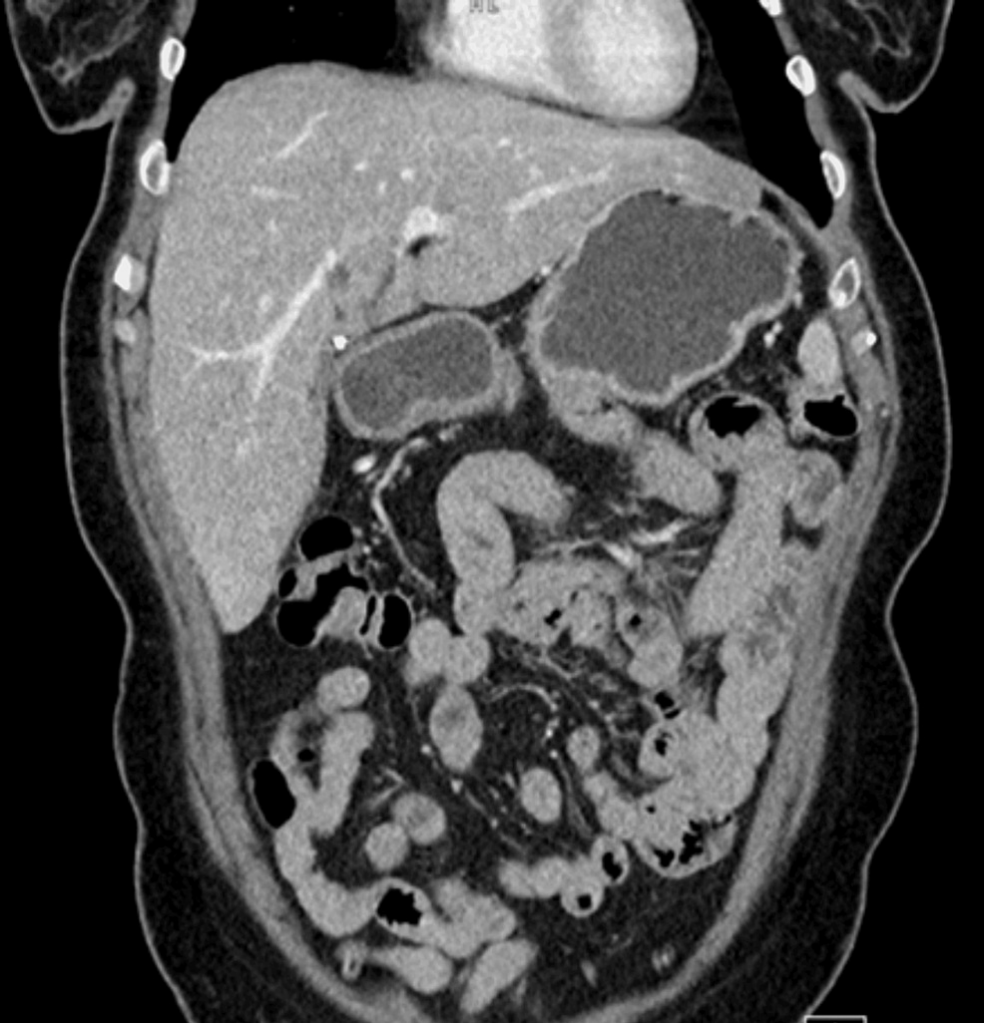
CT of the abdomen and pelvis four months postoperatively showing resolution of the multicystic mass and no evidence of lymphadenopathy.

The patient recovered well and was discharged without complications on postoperative day 4. She was asymptomatic after the one-year follow-up, with the CT of the abdomen and pelvis scan showing resolution of the peritoneal mass and no evidence of lymphadenopathy (Figure 5).

Her CA-125 has remained within the reference range two years after follow-up. Yearly follow-up scans and tumor marker evaluation are planned.

## Discussion

BMPM is a rare, indolent cystic lesion arising from the lining of the peritoneal cavity. The incidence of BMPM is 3 per 2,000,000 per year and most commonly presents in women of reproductive age, although cases involving men and children have been documented [4]. Approximately 200 cases have been reported since the description of the disease in 1928 [4]. The pathogenesis of BMPM has not been elucidated due to the rarity of the disease. It may be attributed to chronic peritoneal irritation, such as from prior surgeries, pelvic inflammatory disease, recurrent peritonitis, and endometriosis [4,6]. This results in chronic inflammation, leading to hyperplasia and dysplasia of mesothelial cells. If such etiology were valid, the pathogenesis of BMPM would be reactive rather than neoplastic. We note that the current patient did not have any previous abdominal surgery or history of peritoneal inflammation. She did carry an uncertain diagnosis of polycystic ovarian syndrome in the chart without a documented supporting aberrant biochemical information.

The tumor’s high recurrence rate after resection and occurrence of cases without chronic peritoneal irritation (like the current patient) are factors that also support the theory of BMPM’s neoplastic origin [3,7,8]. More specifically, one hypothesis suspects that female sex hormones influence mesothelial transformation to BMPM because the disease has a predilection to occur in women of reproductive age. The focal weak staining of estrogen and progesterone receptors in approximately 10% of patients with BMPM also supports this hypothesis [9]. Furthermore, several studies have suggested that BMPM growth depends on estrogen signaling, as oral contraceptives, gonadotropin-releasing hormone (GnRH) agonist, and tamoxifen decreased estrogen levels in some studies and resulted in tumor shrinkage [8,10-11].

The natural history of the disease has not been widely characterized in literature. There are only two known published deaths from BMPM: a 14-year-old patient who refused surgical intervention and a 53-year-old male who died of peritoneal sepsis [12]. Patients with BMPM who elected for surgical resection have been reported to have a good prognosis. However, the lesion has a high recurrence rate of approximately 50%, indicating the need for further intervention. In rare cases, these lesions will also undergo malignant transformation, increasing the risk of mortality.

Common symptoms of BMPM are nonspecific, including abdominal and pelvic pain and tenderness, distension, early satiety, intestinal obstruction, pelvic pain, dysuria, a palpable mass, and unintentional weight loss or weight gain [4]. Several of these symptoms, such as abdominal tenderness, weight loss, and a palpable mass, were identified in our patient. BMPM can be clinically silent, which may contribute to the diagnostic rarity of the disease. BMPM does not have a definite associated tumor serum marker, although elevations in CA 19-9 and CA-125 have been observed [13,14]. Interestingly, our patient had an elevated serum CA-125 prior to surgery, which normalized after resection. It may be a useful tumor marker in this disease process. CEA and CA19-9 were within the reference range prior to surgery.

Imaging modalities such as ultrasound and CT are helpful in identifying thin-walled multiloculated cysts but cannot differentiate BMPM from other diseases with similar characteristics, such lymphangioma, endometriosis, endosalpingiosis, cystic mesonephric duct remnants, adenomatoid tumors, loculated ascites, cystadenoma of the ovary, malignant peritoneal mesothelioma, pseudomyxoma peritonei, and Mullerian cysts of the retroperitoneum [3]. As seen in our patient, ultrasound results were concerning for pseudomyxoma peritonei due to septated fluid. Biopsy of tissues by less invasive tissue sampling modalities, including core needle biopsy and fine needle aspiration, often yields inconclusive findings, as seen in our patient. The most accurate diagnosis is achieved by surgical resection, and histological and immunohistochemical analysis.

**Figure 6:**
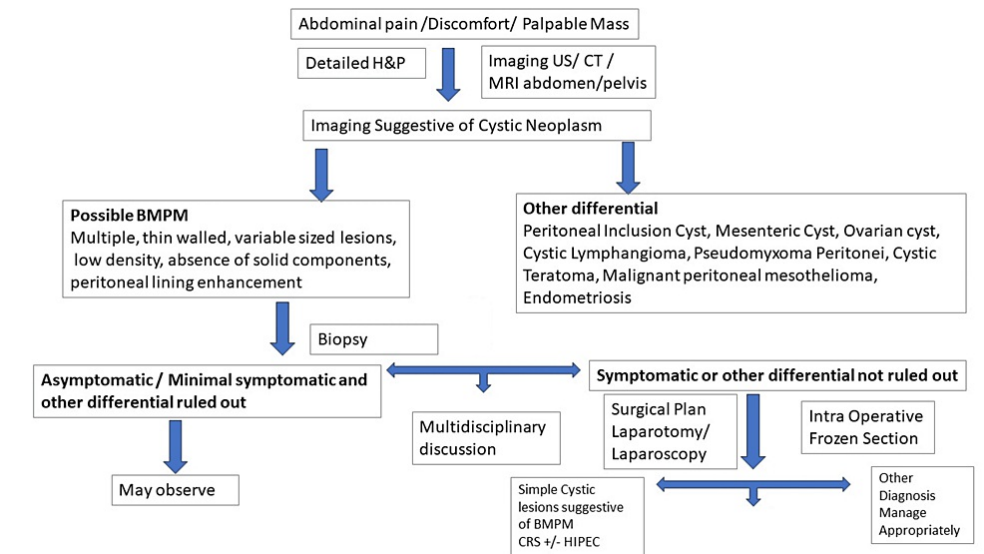
A flow chart describing the key steps and factors to consider in the diagnosis of BMPM. We propose a flow chart that will aid in diagnosing BMPM in patients' with multicystic abdominal lesions. CRS, cytoreductive surgery; BMPM, benign multicystic peritoneal mesothelioma; HIPEC, hyperthermic intraperitoneal chemotherapy; US, ultrasound;

On hematoxylin and eosin stained slides, the morphology criteria of BMPM consisted of a single layer of flat or cuboidal mesothelial cells lining inflamed fibrous septa [7]. There is no atypia, mitosis, or any other characteristics of malignancy. These cysts generally range in size, as seen in our case, with lesions measuring 1.5 cm to 3.2 cm. Calretinin, the marker for mesothelial cells, was used to confirm the presence of mesothelium. The mesothelial cells in BMPM also express Wilms’ tumor antigen, and CK4-7, which is also seen in malignant mesothelioma. BMPM immunohistochemistry is also positive for BAP-1, specifically expressed in adenomatoid tumors, and D2-40, a highly sensitive marker for malignant mesothelioma [7-8,15]. Thus, it is speculated that BMPM is an intermediate condition between adenomatoid lesions and malignant mesothelioma [8]. However, BMPM does not possess homozygous deletion of CKD2NA/B as seen in its’ malignant counterpart. In congruence with the reported literature, the histology of our patient’s lesions showed mesothelial lined cyst, as well as focal areas with adenomatoid proliferation in the stroma with cyst lining cells positive BAP-1 expression. Fluorescence in situ hybridization of CKD2NA/B was negative for homozygous deletion, supporting the benign nature of the disease. Given the complexity of formally diagnosing BMPM, we have developed a flow chart that emphasizes the significant aspects of the work-up (Figure 6).

The management of BMPM does not have a generally accepted evidence-based guideline. Treatment ranges from observation to surgical resection. In asymptomatic patients, observation is commonly recommended as the risks of intervention outweigh the benefits. Symptomatic patients may undergo fine-needle aspiration of the cyst to assess for cytology, followed by sclerotherapy to ablate the fibrinous septa to reduce the rate of recurrence [7]. In addition, symptomatic lesions can also be managed by hormonal modification including oral contraceptives, GnRH agonist such as Lupron, and estrogen antagonist such as tamoxifen, particularly if cytology reveal that the tumor is positive for the estrogen receptors [8-10]. However, non-operative treatment modalities do not accurately reveal the histological characteristics of the tumor.

Surgical resection should be considered in intraperitoneal cystic neoplasms, if malignancy cannot be ruled out. It has been reported that surgical resection alone of BMPM leads to around 50% of recurrence. Addition of HIPEC is superior to surgical resection alone as the recurrence rate of the disease decreases to 0%-21% [11,16-17]. The surgeon and the institution should be trained, equipped, and planned to administer this therapy. Hence, surgeons should consider CRS and HIPEC in all patients with indeterminate multicystic peritoneal lesions. Irradiation and adjuvant chemotherapy have not been shown to be effective [11].

## Conclusions

The etiology of BMPM is still poorly understood, as there is not enough evidence to show whether the lesion arises from a neoplastic or inflammatory process. Due to the rareness of the disease, there is no generally accepted treatment modality for definitive care. In this case report, we highlight the symptoms, the challenges of accurate preoperative diagnosis, treatment, and surveillance of BMPM. The management of BMPM with surgical resection alone does possess a high recurrence rate, thus we highlight the importance of HIPEC at the index operation. Further research is required to elucidate the optimal management strategy.
